# Clustering patient mobility patterns to assess effectiveness of health-service delivery

**DOI:** 10.1186/s12913-017-2381-2

**Published:** 2017-07-04

**Authors:** Selman Delil, Rahmi Nurhan Çelik, Sayın San, Murat Dundar

**Affiliations:** 1 Informatics Institute, Istanbul Technical University, Istanbul, Turkey; 2 Department of Financial Econometrics, Sakarya University, Sakarya, Turkey; 3 Department of Computer & Information Sciences, Indiana University-Purdue University, Indianapolis, IN USA

**Keywords:** Patient mobility, Clustering patient mobility, Health-service delivery, Turkish health-care system, Hierarchical clustering, Gandy nomogram

## Abstract

**Background:**

Analysis of patient mobility in a country not only gives an idea of how the health-care system works, but also can be a guideline to determine the quality of health care and health disparity among regions. Even though determination of patient movement is important, it is not often realized that patient mobility could have a unique pattern beyond health-related endowments (e.g., facilities, medical staff). This study therefore addresses the following research question: Is there a way to identify regions with similar patterns using spatio-temporal distribution of patient mobility? The aim of the paper is to answer this question and improve a classification method that is useful for populous countries like Turkey that have many administrative areas.

**Methods:**

The data used in the study consist of spatio-temporal information on patient mobility for the period between 2009 and 2013. Patient mobility patterns based on the number of patients attracted/escaping across 81 provinces of Turkey are illustrated graphically. The hierarchical clustering method is used to group provinces in terms of the mobility characteristics revealed by the patterns. Clustered groups of provinces are analyzed using non-parametric statistical tests to identify potential correlations between clustered groups and the selected basic health indicators.

**Results:**

Ineffective health-care delivery in certain regions of Turkey was determined through identifying patient mobility patterns. High escape values obtained for a large number of provinces suggest poor health-care accessibility. On the other hand, over the period of time studied, visualization of temporal mobility revealed a considerable decrease in the escape ratio for inadequately equipped provinces. Among four of twelve clusters created using the hierarchical clustering method, which include 64 of 81 Turkish provinces, there was a statistically significant relationship between the patterns and the selected basic health indicators of the clusters. The remaining eight clusters included 17 provinces and showed anomalies.

**Conclusions:**

The most important contribution of this study is the development of a way to identify patient mobility patterns by analyzing patient movements across the clusters. These results are strong evidence that patient mobility patterns provide a useful tool for decisions concerning the distribution of health-care services and the provision of health care equipment to the provinces.

## Background

As basic human rights, medical care and necessary social services must be accessible for people [1]. The principle of territoriality has become essential to the organization and consumption of health care to improve health-care access and utilization management [2]. However, regional disparities can still arise from geographic differences, limited human and financial resources, and other constraints that make for unequal distribution of resources among health administrative areas. Regional disparities in the quality of health-care services eventually lead to patient mobility.

Today, people can access health information more quickly than ever before and have higher expectations of quality and performance from health-care services. Social security systems providing many alternatives also give patients the opportunity to move across regions, countries, and even continents. For instance, in Europe several rulings made by the EU Court of Justice extended the cross-border care right of EU citizens [3, 4]. In 2011 the EU Parliament adopted a directive that allows EU citizens to be treated in any other EU country [5].

Analysis of patient mobility can play a key role toward better understanding of health-care systems by decision makers and can offer additional insight into identifying medical and management areas that need capacity improvement to satisfy health-care needs. Patient mobility can also be considered as an important parameter to observe the impact of reforms, legislative changes, and other developments in a health-care system.

Given the importance of patient mobility, the aim of this paper is not only to analyze patient mobility across Turkey, but also to develop a method that improves the ability of decision makers to access these mobility patterns. The paper will focus on the classification of mobility characteristics of different health-care areas (e.g., provinces, regions, or states) to identify patient mobility patterns. Thus, one can statistically test for a significant relationship between clusters that are based on patient mobility patterns with a comprehensive spatial and temporal perspective. Other research questions are as follows:

- Are there any meaningful differences in patient mobility across health-service areas with respect to spatial and temporal variations?
- Can the clustering of health-service units based on patient mobility be independently explained using basic health-care indicators?

Patient mobility is usually categorized as intra-regional (within a region) or extra-regional (out of a region) for study of mobility within a certain region, or interregional (between regions) for study on a national scale [6, 7]. International mobility, cross-border mobility, or health tourism are some of the terms used for country-to-country health travel [3, 8, 9]. This study covers the range of interregional patient mobility previously studied in Spain and Italy to improve the understanding of health economics [6, 7, 10–15]. In these countries, as in Turkey, local health departments have gained more autonomy in the past few decades, while central governments still maintain regulatory roles.

In 2011 the Turkish Ministry of Health (TMoH) released a new region-centered planning document for required health services [16]. In this regulation, 81 provinces were partitioned into 29 health regions with one province from each region identified as the regional health-care center. According to the policy adopted in this document, patients in each region should first seek medical care from their regional health-care center before considering other centers. Under these new arrangements and the previously adopted health transformation program, the number of patient admissions to hospitals has increased dramatically. Turkey has a three-level health system – primary care (family physicians), public and private hospitals (specialist physicians), and training and research hospitals – and in this study, we are mainly going to consider the second two.

Some studies have addressed patient mobility in general using different sampling units, parameters chosen, and methods employed (e.g., panel data analysis, econometric regression models, and gravity models). Most have confirmed the relationship between patient mobility and health-care service delivery (quality, productivity, infrastructure, location). For instance, using panel data analysis Mafrolla and D’Amico [15] found a strong correlation between patient mobility behavior and health-care efficiency. In addition, certain studies have been conducted on specific issues, such as pediatric diseases [6], aortic valve substitution [7], percutaneous transluminal coronary angioplasty (PTCA) [10], or cardiac surgeries stratified according to severity levels [11], using patient-mobility parameters.

Unlike studies that focus on examining the motives leading to patient mobility, this paper analyzes the similarities and differences between cluster patterns based on patient mobility across Turkish provinces and will contribute to the literature by suggesting a way for identifying patient mobility patterns that indicate quality and accessibility of health-care services. Furthermore, this paper is unique in considering patient mobility patterns among all provinces of Turkey. Other papers have considered health-care authorities in a certain region [15], or at most 21 regions on a national scale [14].

As a practical analytical tool for comparing access across many geographical areas in a single presentation, Gandy’s nomogram (the Nomogramma di Gandy: NdiG) can be used to explore clusters related to access to health-service areas [17, 18]. NdiG is a special graph that plots mobility parameters of service areas (i.e., pairs of attraction and escape values) in a Cartesian space. In this study, NdiG will be used to create a tool that identifies patient mobility patterns and clusters.

The paper is organized as follows: The following heading briefly reviews the Turkish health-care system. Then, we presented methods on mobility parameters, data description and preparation, Gandy’s graphic demonstration, and clustering mobility patterns. In the Results and Discussions heading, we discussed results of clustering and statistical tests along with examples of temporal mobility patterns identified. Lastly, we provided conclusions and ideas for future work.

## The Turkish health-care system

Health services in Turkey are organized across the 81 provinces under the control of the Provincial Health Directorates (PHDs), which are managed by the Turkish Ministry of Health. At the local level, PHDs plan and provide health services, coordinating with the newly established Public Health Directorate (PuHD) and the Association of Public Hospitals (APH). As supervisor of community health centers (CHCs) and family health centers (FHCs), the PuHD provides primary health services. The APH is the management organization for public hospitals in a province. The PHDs are also responsible to their provincial governors, who represent the government [19].

Health providers in Turkey are organized into three levels. At the first level are the primary health centers staffed by family physicians, at the second level are public and private hospitals, and at the third level are training and research hospitals (TRHs) managed by APH and independent university hospitals.

The Turkish Healthcare System (THS) has changed radically in the past decade following the initiation of the Turkey Health Transformation Program (THTP) in 2003. This governmental program aimed to improve governance, efficiency, and quality in the health-care sector [20]. THTP has been active in the health system through significant investment in the hospital sector and the establishment of a family-physician system [21].

Public- and private-sector employees were combined under the newly created Turkish Social Security Institution (TSSI), and almost the entire population is covered by social insurance with universal health coverage. After many legislative changes, TSSI has become the sole buyer of health-care services, while the TMoH has become the main health-care service provider in the country [22]. With the implementation of THTP in Turkey, citizens are now free to choose treatment in either a private or a public health center, without any referral requirement. According to an OECD health-care report, THTP has been successful in prioritizing coverage, access, and activity, and the focus of the THS should now shift to quality and outcomes [21].

## Methods

### Mobility parameters

Patient mobility can be defined as the movement of a patient to another region/place to seek better health-care service. The key variable is whether patients receive treatment in the place in which they reside or in another place. The number of patients “attracted to” and “escaping from” provinces is extracted from the data and used to explain patient mobility relationships among the provinces. These indicators have been employed in different formats in previous studies, for instance by normalizing them according to population or intra-regional mobility [7, 11–15]. Attraction is determined by the number of patients coming from other provinces to receive health-care services; escape measures the number of patients traveling to other provinces for the same purpose. Patient mobility variables used in this study are shown below:

**Attract:** Number of patients coming from other provinces.

**Escape:** Number of patients traveling to other provinces.

**Resident Admissions:** Number of patients admitted from the same province as the center.

**TPA:** Total patient admission in a province (Resident Admissions + Attract)

**TRP:** Total resident patient population in a province (Resident Admissions + Escape)

### Data

The data were obtained from the TSSI, following required legal procedures and protecting patient and institutional privacy. The data contain the number of patients admitted to health-care centers on a yearly basis. The study covered the 4 years beginning December 2009 and ending December 2013.The data set contains information on more than 1.2 billion hospital admissions occurring during this time period. The admission records were summarized with respect to provinces.

Yearly mobility ratios in Table 1 suggest that the patient mobility ratio is declining year by year in Turkey. Location information was not available for 11% of patients, whose citizenship ID was not available in the National Address Database or who were non-citizens coming for cross-border medical tourism. These records were taken into account when calculating attract ratios for each province, as patient origin is not relevant for computing the attract ratio.

**Table 1:** Hospital admission and mobility by year

Term	Total Hospital Admissions	Mobility	Mob. Ratio
Dec.2009-Nov.2010	251,630,100	32,843,706	13.05%
Dec.2010-Nov.2011	292,626,833	36,407,051	12.44%
Dec.2011-Nov.2012	355,843,020	41,755,845	11.73%
Dec.2012-Nov.2013	372,586,211	43,772,750	11.75%

This study focuses mainly on secondary and tertiary care and ignores primary care. Owing to recent improvements in the Turkish health-care system almost all patients in Turkey can now receive quality primary care in their home town. In additional, emergency admissions and some medical-specialties such as environmental health, army medicine, and occupational medicine have also been ignored, as these types of admissions can bias general mobility information.

### Gandy’s graphic demonstration

**Fig. 1:**
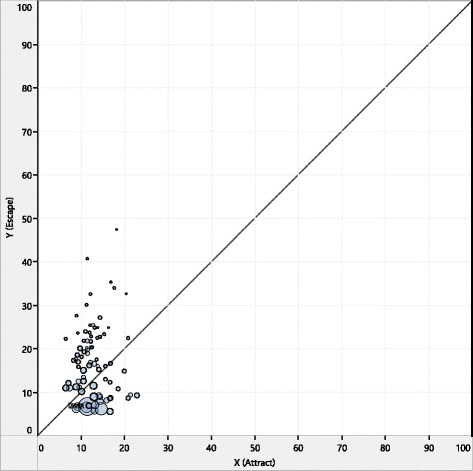
Plotting patient mobility with Gandy nomogram (average of 2010–2013)

To compare mobility characteristics and seasonal fluctuations for provinces, NdiG graphic demonstrations are used as a practical analytic tool that allows comparison of regions in terms of attract and escape values to show accessibility of public services [17]. The NdiG was enhanced by adding visualization properties and an automatic timeline view. A graphic interface developed in Tableau software [23] can automatically calculate mobility parameters with respect to time**.** In this graphic interface, nodes represent provinces and the diagonal line represents equality between escape and attract ratios (Fig. 1).

While different studies use different formulations to calculate the NdiG axes, the following formulas were employed to produce normalized attract and escape ratios for each province:

$$ \mathrm{Attract}\ \mathrm{ratio}\ \left(\mathrm{X}\ \mathrm{axis}\right)=\frac{\mathrm{Attract}}{\mathrm{TPA}}\ast 100 $$

$$ \mathrm{Escape}\ \mathrm{ratio}\ \left(\mathrm{Y}\ \mathrm{axis}\right)=\frac{\mathrm{Escape}}{\mathrm{TRP}}\ast 100 $$

On the coordinate plane, increase in attract ratio is measured along the X axis, increase in escape ratio along the Y axis.

### Clustering mobility patterns

In order to identify similarities and differences among provinces, the patient mobility data involving attract/escape pairs were clustered using the agglomerative hierarchical (linkage) clustering method [24]. This method calculates the distance between every pair of feature vectors in the data set and moves up the hierarchy by merging pairs of clusters. Compared to other clustering algorithms, hierarchical clustering is more suitable for our task as one can choose a cut-off level by dynamically observing the hierarchy being formed, in this case among provinces.

For each of the 81 provinces, quarterly attract/escape values were computed and averaged over 4 years to obtain an 8-dimensional feature vector (two features for each quarter). The agglomerative hierarchical clustering algorithm was executed on the resulting 81-by-8 feature matrix. Several different distance metrics (Manhattan, Cosine, Euclidean) were used to test the calculation of distance between observations. After the distance between objects is computed, a linkage function is calculated to group objects into clusters. Three popular linkage methods (single, complete, average) were used with the agglomerative hierarchical clustering algorithm. The cophenetic correlation coefficient [24, 25] was calculated to compare the linkage distances between clusters to the original distance matrix. This coefficient determines correlation between two distances. Table 2 includes correlation coefficient results for all combinations of distance metrics and linkage methods.

**Table 2:** Cophenetic correlation coefficient results

Distance Metric	Linkage Method	Cophenetic Correlation Coefficient
Manhattan (Cityblock)	Single	0.7333
	Complete	0.6830
	Average	0.7693
Cosine	Single	0.7016
	Complete	0.6887
	Average	0.7551
Euclidean	Single	0.7016
	Complete	0.6783
	Average	0.7863

These results suggest that the Euclidean distance metric with an average linkage method produces the highest correlated coefficient (*r* = 0.7863). The Euclidean method measures the distance between two observations by the length of the path directly connecting them.

Given that *X* is an *n* × *d –*dimensional data matrix in which each row corresponds to a *d-dimensional* point *in Euclidean space* for each observation, to calculate the distance between two points *X*_*a*_ and *X*_*b*_ the following equations are used:

$$ {X}_a=\left[{a}_1\kern0.5em {a}_2\kern1em ..\kern0.5em {a}_d\right] $$

$$ {X}_b=\left[{b}_1\kern0.5em {b}_2\kern1em ..\kern0.5em {b}_d\right] $$

$$ {d}_{euclidean}\left({X}_a,{X}_b\right)=\sqrt{{\left({a}_1-{b}_1\right)}^2+{\left({a}_2-{b}_2\right)}^2+\dots +{\left({a}_d-{b}_d\right)}^2} $$

## Results and Discussions

Both demand-side (e.g., regional income disparities, patient preferences, health status, beliefs) and supply-side (e.g., health-provider financial incentives, ability or practice norms) factors can cause regional variations among health-care service areas [26]. On the other hand, as we mentioned in the first section, correlation between patient mobility and health-care service parameters (e.g., quality, efficiency, infrastructure, location) have previously been confirmed in many studies [6, 7, 10, 11, 15]. Therefore, we consider the changes in patient mobility parameters (attract/escape) as proxies for availability and accessibility of health-care services.

### General mobility patterns on the NdiG

Figure 1 shows the distribution of 4-year (2010–2013) average mobility pairs for each province. This figure shows that escape rates spread out more than attraction rates. No province has an attraction rate greater than 23, whereas 18 provinces have escape rates exceeding this value. This distribution of attract/escape pairs can be considered as a confirmation of specialized regional health-care service delivery [16] and reflects the inevitable geographic location disadvantage of some eastern settlements in Turkey. However, a high escape value for a large number of provinces might be an indication of poor health-care accessibility arising from unequal distributions of central health-service locations among provinces of Turkey.

Another interesting observation is that provinces with high TPA values (node sizes are adjusted according to the TPA) are grouped at the bottom left corner of the graphic (i.e., between 5 and 10 escape, and 8 and 17 attract intervals). Provinces with high escape values lie on the upper left side of the diagonal line. These results are further discussed in Clustering results.

### Analysis of temporal patient mobility patterns

**Fig. 2:**
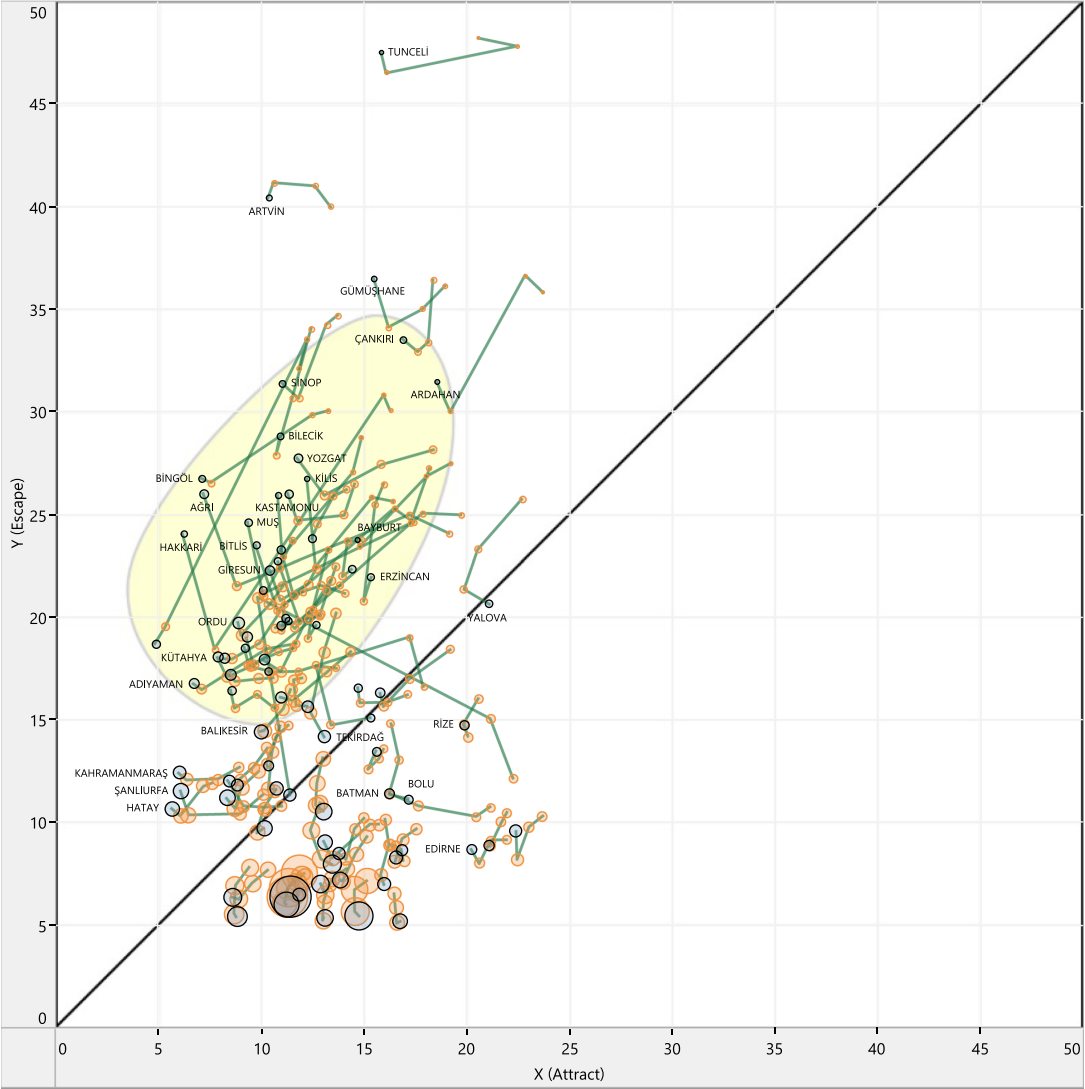
General landscape of yearly mobility patterns (2010–2013)

**Fig. 3:**
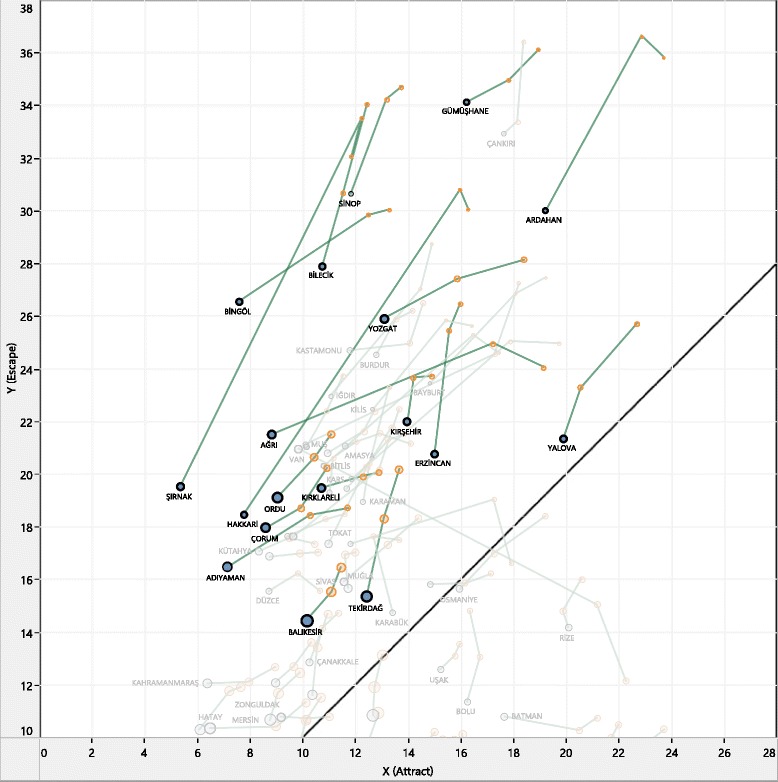
Selected trend patterns of yearly mobility patterns

**Fig. 4:**
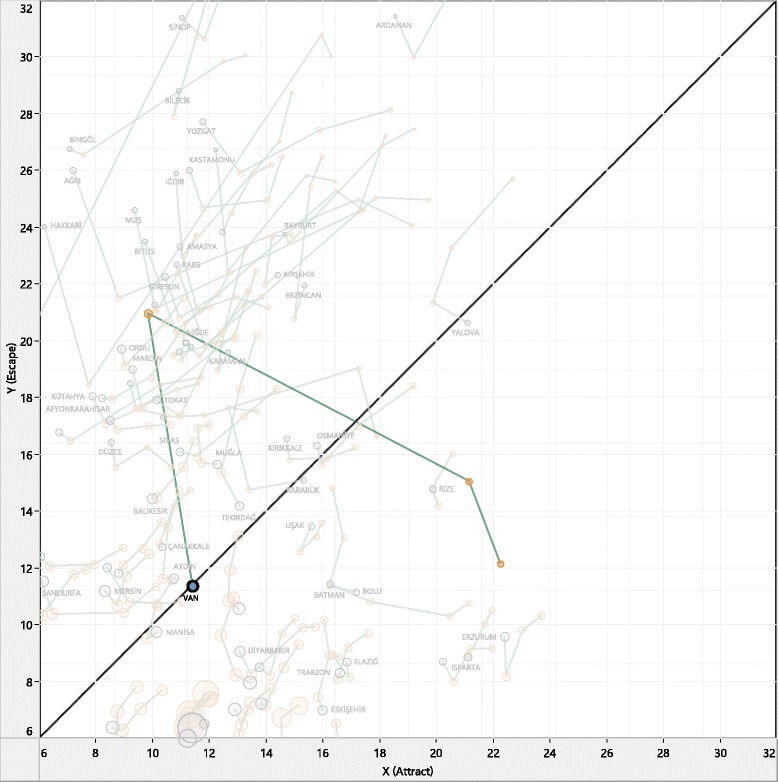
Temporal escape: Van earthquake (Oct. 2011)

Temporal mobility visualization can provide valuable insight on how patients react to changes in the health-care system, especially when we look at the yearly changes. The temporal patterns generated based on yearly changes of attract/escape values were analyzed on NdiG graphs to show mobility trends (Figs. 2, 3 and 4). To observe temporal patterns, a broken-line graph was drawn for each sequence of data points (i.e., a 4-year time series for the mobility pairs of each province). To follow fluctuations and mobility direction on each line-graph, observation history (e.g., the first 3 years) was colored with an orange hue, and the last node was colored with blue. The first orange node on each line-graph indicates the starting point of the time-series, which is 2010, and the blue node at the end of the line represents the most recent year (i.e., in Fig. 2 2013, in Fig. 3 2012).

Figure 2 shows the general landscape over the 4-year period studied and Fig. 3 shows selected patterns in the shaded region of Fig. 2. It can be observed from Fig. 2 that yearly mobility pairs seem to have similar trends, especially for provinces with high escape values. When we look more closely at the selected patterns in Fig. 3, most of the broken-line graphs drawn between observations have positive slope, indicating a considerable decrease in both attract and escape parameters corresponding to these provinces. This means that the more recent years have lower escape and attract ratios at the same time. This movement on the graph matches well with considerable improvements in THS for this period. At the end of 2010, the Family Medicine Programme, assigning each patient to a specific doctor, was established throughout the country [20] and a considerable number of physicians in different specialties were assigned to the disadvantaged provinces in this period.

To confirm the observation on the graph, statistical tests were performed to determine if these changes in attract/escape parameters over the years are statistically significant. We tested whether the mean of the differences between two paired samples differs. The parameter values corresponding to the first (2010) and last (2013) years were compared for each province. The attract/escape pairs were tested by a paired t-test and demonstrated a statistically significant (Esc. *p*-value = 0.000, Att. *p*-value = 0.000) relationship between pairs. Descriptive statistics and test results are summarized in Table 3.

**Table 3:** Statistical test for mobility variations

	Esc2010	Esc2013	Att2010	Att2013
Min.	0.0652	0.0518	0.0763	0.0484
Max.	0.4818	0.4748	0.237	0.224
Median	0.1737	0.1644	0.1385	0.1129
Mean	0.1881	0.1705	0.1473	0.121
Var.	0.00814	0.0075	0.00142	0.00147
Std.dev.	0.09026	0.08665	0.03774	0.03835
Paired T-Test Results	*t* = 7.4165, df = 80, *p*-value = 1.113e-10		*t* = 8.659, df = 80, *p*-value = 4.11e-13	

A decreasing escape ratio, which generally occurs for ratios that are high to begin with, would seem to indicate improving health-care opportunities for a disadvantaged region. Although patients continue to travel for specialized treatments owing to the fact that new facilities may not offer secondary and tertiary care or might not have sufficient health staff, proportionally they travel less compared to the past. The fact that the attract ratios for these points are decreasing simultaneously confirms the general positive trend throughout THS under implementation of the THTP program. As mentioned previously, one consequence of developments in health policies in the THS (e.g., universal health coverage) has been a dramatic increase in patient admissions. This could also affect mobility indicators by scaling down their proportion to total admissions (i.e., TPA).

Patient mobility in Van, a major province in Eastern Anatolia, exhibits a unique pattern and deserves examination (Fig. 4). In 2011, a major earthquake hit the city of Van, killed over 500 people, and caused thousands to flee to other cities. Although all demolished infrastructure, including hospitals, was mostly rebuilt in less than a year, the results suggest that attraction lost owing to the earthquake had not been fully recovered as of 2013.

### Clustering results

**Fig. 5:**
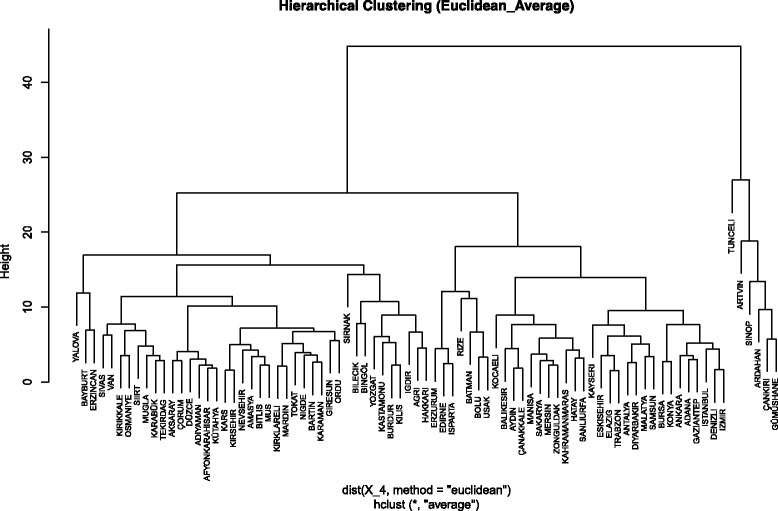
Dendrogram plotting for linkage clustering

Figure 5 shows a dendrogram generated using the results obtained by the agglomerative hierarchical clustering algorithm. The cut-off level is chosen in an empirical way so that general knowledge about provinces can be incorporated into the clustering process. Various cut-off values were tested and a value of 12 was chosen after consideration of cluster members and cluster size distributions in each case. This cut-off is selected so as to reconcile the output of the automated clustering with the domain knowledge the authors have about the 81 provinces-- the economic welfare of the provinces is taken into account together with the distances in the dendrogram to determine an empirical cut-off height to produce the clusters.

**Fig. 6:**
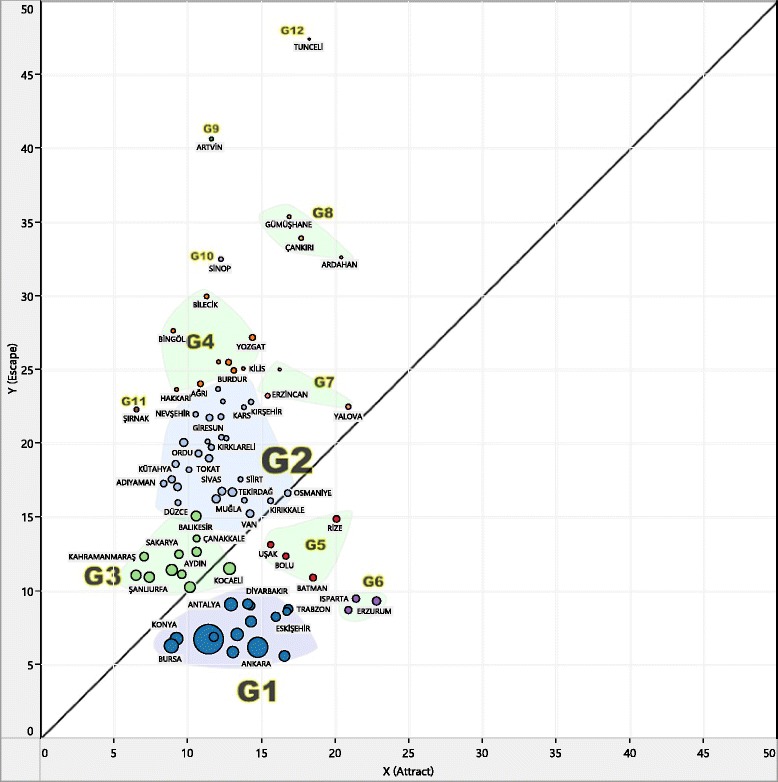
Plotting of clustering results on large layout

Provinces that fall into each cluster are shown in Table 4. Twelve clusters were established, four of which (G1-G4) contain 64 of the 81 provinces. Color labeling was used for these clusters on the NdiG graph in Fig. 6. It is no surprise to see that the most-populated provinces, including Istanbul, Ankara, and Izmir, are in the same cluster at the bottom of the diagram (G1), while the least-populated provinces with high escape ratios generally show in clusters above the diagonal line and away from the bottom left-hand corner of the graph (e.g., G2, G4, G7). However, some provinces do not fit into any of these easily identifiable groups. Edirne, Erzurum, and Isparta, which have the highest attract values among the 81 provinces as well as moderately low escape values, are grouped together (G6), suggesting that these three provinces have similar patient mobility characteristics that differ from other dominant groups.

**Table 4:** Cluster memberships of 81 provinces

Grp:01 (n: 16)	Grp:02 (n: 28)	Grp:03 (n: 11)	Grp:04 (n: 9)	Grp:05 (n: 4)
Adana, Ankara, Antalya, Bursa, Denizli, Diyarbakır, Elazığ, Eskişehir, Gaziantep, İstanbul, İzmir, Kayseri, Konya, Malatya, Samsun, Trabzon	Adıyaman, Afyonkarahisar, Aksaray, Amasya, Bartın, Bitlis, Çorum, Düzce, Giresun, Karabük, Karaman, Kars, Kırıkkale, Kırklareli, Kırşehir, Kütahya, Mardin, Muğla, Muş, Nevşehir, Niğde, Ordu, Osmaniye, Siirt, Sivas, Tekirdağ, Tokat, Van	Aydin, Balıkesir, Çanakkale, Hatay, Kahramanmaraş, Kocaeli, Manisa, Mersin, Sakarya, Şanlıurfa, Zonguldak	Ağrı, Bilecik, Bingöl, Burdur, Hakkari, Iğdır, Kastamonu, Kilis, Yozgat	Batman, Bolu, Rize, Uşak
Grp:06 (n: 3)	Grp:07 (n: 3)	Grp:08 (n: 3)	Grp: 09 - ArtvinGrp: 10 - SinopGrp: 11 - Şırnak Grp: 12 -Tunceli	
Edirne, Erzurum, Isparta	Bayburt, Erzincan, Yalova	Ardahan, Çankırı, Gümüşhane

Additionally, Batman, Rize, Bolu, and Usak, all of which are moderately populated provinces with considerable attract ratios, are grouped in the same cluster. Furthermore, abnormal provinces such as Sırnak (G11) were identified and presented as isolated clusters. Although Sırnak is in an economically disadvantaged part of Turkey, it has had considerable development in its health-service infrastructure and capacity, which resulted in a significant increase in the number of hospital beds (both public and private) available and specialist physicians practicing during the period studied [27].

### Statistical analysis of clustering results

After clustering provinces by their patient mobility characteristics, the next step is to identify independent parameters associated with individual provinces that would best explain these clustering results. The statistical tests can provide further confirmation of the clustering output using independent health-care parameters and their population-adjusted versions and offer a more convincing case as to the predictive power of mobility features (attract and escape) in explaining health-care service quality and health infrastructure.

For this purpose, the following variables were considered: province population, number of health personnel in the province (physicians, specialists, total number of physicians/specialists, and other health personnel), number of beds (public, private, and university), and their population-adjusted versions. The related health indicators for each province were derived using data released by the TSI [27] for the period 2010–2013. Four-year average values for each province were used and these values were averaged over all provinces within each cluster to obtain health indicators for each cluster (Table 5).

**Table 5:** Average values for selected health indicators

Clusters	No. of Members	Population	Specialist Physicians		Physicians		Total Physicians		Other Health Personnel		Total Hospital Beds		University Beds		Private Beds	
		A	A	B	A	B	A	B	A	B	A	B	A	B	A	B
1	16	2,575,993	3069	114	1058	46	4127	160	8582	396	6850	296	1562	78	1368	44
2	28	474,626	649	133	337	73	986	206	2685	589	1069	228	96	17	119	22
3	11	1,190,143	832	73	525	46	1358	118	3879	342	2420	215	291	26	289	23
4	9	298,027	278	106	218	81	496	188	1684	642	556	184	6	1	43	12
6	3	531,406	930	187	378	71	1308	259	3332	664	2409	462	862	166	137	29
5	4	368,144	512	156	292	85	804	241	2496	737	1069	312	61	22	190	46
7	3	168,572	243	138	186	111	429	249	1464	891	342	201	0	0	44	20
8	3	141,046	183	150	147	116	330	266	1126	878	311	215	0	0	26	14
9	1	166,892	357	214	214	128	572	342	1915	1147	471	282	0	0	0	0
10	1	202,912	273	135	239	118	512	252	1602	790	531	262	0	0	0	0
11	1	457,586	149	33	204	45	353	77	915	200	490	107	0	0	0	0
12	1	83,366	325	389	261	313	586	702	1418	1700	177	212	0	0	0	0
Total	81	928,218	1063	125	473	73	1537	197	3752	574	2300	246	417	32	372	26

A: Cluster Average for Total Number

B: Cluster Average per 100,000 Population

Non-parametric statistical tests were performed to determine if these parameters are significantly correlated with the clustering results. Descriptions of parameters used in statistical tests are included in Table 6. Kruskal-Wallis (rank-based) [28] statistics were used to explain mean differences between groups. The Kruskal-Wallis (K-W) is considered the non-parametric equivalent of the analysis of variance (ANOVA) test. This test is most appropriate when the response variable is categorical and the level of measurement is continuous. Small clusters (G5-G12) were excluded from statistical tests mainly because the K-W statistical test requires at least six samples for each group. However, all clusters were included in the overall analysis of the results in the prior section.

**Table 6:** Description of variables for statistical analysis

Variable^a^	Description	Types of Variable
Cluster Groups	Clustering results of patient mobility data	Categorical
Population	Population of the cities	Continuous
S_Physi	Number of specialist physicians (medical residents are considered as specialists)	Continuous
Physi	Number of medical practitioners	Continuous
T_Physi	Total number of physicians	Continuous
Other_Per	Total number of other health-care personnel in the city	Continuous
T_B	Total number of hospital beds in the city	Continuous
Prv_B	Number of hospital beds in private health centers in the city	Continuous
Univ_B	Number of hospital beds in university health centers in the city	Continuous
S_Physi_Pop	(S_Physi / Population) ^a^ 100,000	Continuous (Ratio)
T_Physi_Pop	(T_Physi / Population) ^a^ 100,000	Continuous (Ratio)
Other_Per_Pop	(Other_Per / Population) ^a^ 100,000	Continuous (Ratio)
Prv_B_Pop	(Prv_B / Population) ^a^ 100,000	Continuous (Ratio)
Univ_B_Pop	(Univ_B / Population) ^a^ 100,000	Continuous (Ratio)
TB_Pop	(T_B / Population) ^a^ 100,000	Continuous (Ratio)

^a^All variables are averages over 4 years except the cluster groups

The *p*-values in Table 7 suggest that there is a statistically significant difference between groups with respect to all but one parameter (T_Physi_Pop). The results imply that the major clusters of provinces identified based on patient mobility data have statistically significant correlation with the corresponding basic health indicators of each cluster, with the population-adjusted total number of physicians being the only exception. These results suggest that patient mobility can be used as a proxy for health-care parameters to evaluate overall effectiveness of health-service delivery in a country, with the additional advantage of identifying anomalies or gaps in the system that would otherwise go undetected using health parameters alone.

**Table 7:** Kruskal-Wallis test results

Variable	Chi-squared	*P*-Value(df = 3)
Population	41.3552	5.498e-09
S_Physi	34.2144	1.785e-07
Physi	31.198	7.722e-07
T_Physi	33.9442	2.036e-07
Oter_Per	33.5121	2.511e-07
T_B	45.9917	5.695e-10
Univ_B	44.6966	1.073e-09
Prv_B	38.2541	2.497e-08
S_Physi_Pop	10.4101	0.01538
Physi_Pop	18.6265	0.0003266
T_Physi_Pop	5.0931	0.1651
Other_Per_Pop	8.1789	0.04245
TB_Pop	13.7041	0.003337
Univ_B_Pop	33.7183	2.272e-07
Prv_B_Pop	21.31	9.077e-05

## Conclusions

Considering the research questions emphasized in the first section, this study shows the importance of analysis of patient mobility for comprehensive understanding of service-delivery differences across different geographical regions of a country. Furthermore, this paper fills the research gap in the literature on classification of regions based on patient mobility, especially when an increasing numbers of health administrative areas (as is the case in Turkey) and temporal trends are taken into consideration.

In this study, provinces with similar patterns of patient mobility were identified using the agglomerative hierarchical clustering algorithm. Four major clusters were obtained plus several smaller and isolated ones. Statistical tests show that groups identified by clustering patient mobility data correlate, in a statistically significant manner, with all but one of the basic health-care indicators considered. Furthermore, ineffective health-care delivery in certain regions of Turkey was determined through identifying patient mobility patterns.

The primary contribution of the study is the methodology that allows decision makers to identify patient mobility patterns and determine inequality in the distribution of health-care services. Although Turkish patient mobility data were used in the paper, this type of analysis of the patterns between clusters can be applied to other countries. The methodology used can be a guideline for administrators to identify potential gaps in existing health-care services and can play an important role in future planning decisions concerning improvement in the quality of health-care delivery in disadvantaged provinces as well.

Multiple groups of disadvantaged provinces with high escape ratio were identified; patients in these provinces have to make considerable effort to reach centralized health services. On the other hand, there were also groups of provinces with close to ideal balance (i.e., they are close to the bottom left-hand corner) in terms of patient mobility. For possible future planning decisions, those disadvantaged provinces can receive more investment in health infrastructure to provide ideal public service delivery, while the others can advance in specialized health services.

Moreover, the results of clustering show that the attraction ratio of certain provinces in terms of patient mobility does not depend on health-related services and socioeconomic characteristics alone. Even though provinces such as Batman, Usak and Bolu have fewer health-related facilities and lower socioeconomic status, their attraction levels are comparatively higher than usual among small and medium-sized provinces. Non-health-related variables such as kinship among province populations or the recognition of specialists in these provinces must also be considered. Further studies should analyze the relationship between patient mobility and non-health-related variables.

In this research, analysis of patient mobility was limited to Turkish health-care delivery at the national level irrespective of any medical specialty. However, the clustering approach in patient mobility analysis can also be applied at the medical-specialty level to identify clusters of health-service regions relatively more (or less) attractive for a given medical specialty. This will provide an opportunity to better understand regional accessibility of health-care service for a specific branch that would not be possible from the overall analysis of the data. For further studies, a graphical user interface for visualizing spatio-temporal relationships among regions would be more efficient for identifying correlations between health indicators and patient mobility.

## Abbreviations

APH: Association of Public Hospitals
CHCs: Community health centers
EU: European Union
FHCs: Family health centers
NdiG: Nomogramma di Gandy
PHDs: Provincial Health Directorates
PuHD: Public Health Directorate
THS: Turkish Healthcare System
THTP: Turkey Health Transformation Program
TMoH: Turkish Ministry of Health
TPA: Total patient admission in a province (resident admissions + attract)
TRHs: Training and research hospitals
TRP: Total resident patient population in a province (resident admissions + escape)
TSSI: Turkish Social Security Institution
